# Club foot (talipes equinovarus) and ipsilateral infantile hemangiomas: Evaluating a possible connection

**DOI:** 10.1016/j.jdcr.2026.04.025

**Published:** 2026-04-21

**Authors:** Cristina M. Foschi, Leah Lalor

**Affiliations:** a Department of Dermatology, Medical College of Wisconsin, Milwaukee, Wisconsin

**Keywords:** club foot, LUMBAR syndrome, partial segmental hemangioma, talipes equinovarus

## Introduction

Infantile hemangiomas (IHs) on the lower body, particularly those which are segmental, can be associated with underlying anomalies. In particular, lower body hemangioma, urogenital abnormalities/ulceration, myelopathy, bony deformities, anorectal malformations and arterial anomalies, renal anomalies (LUMBAR) syndrome describes the connection between these hemangiomas and urogenital anomalies, ulceration, spinal cord malformations, bony deformities, anorectal malformations, arterial anomalies, and renal abnormalities.^1^ While SACRAL syndrome, an abbreviation which partially describes these features of spinal dysraphism, anomalies of anogenital, cutaneous, renal and urologic origin, and angioma of lumbosacral localization, has previously been established, LUMBAR is considered to be the most comprehensive acronym.^1^ Depending on the location of the hemangioma, certain anomalies may be more common than others. Bony defects have been reported in approximately 20% of published LUMBAR syndrome cases, with sacrococcygeal dysplasia being the most frequently reported bony anomaly.^1^ Talipes equinovarus, or club foot, is another musculoskeletal birth defect reported in LUMBAR syndrome, although not a particular entity included in its diagnostic criteria. While club foot is a common bony anomaly seen in infants overall, its report in LUMBAR syndrome may suggest a possible association between the development of an IH and musculoskeletal formation. Although bony deformities have been reported in LUMBAR syndrome with extensive segmental IHs, the potential relationship between a musculoskeletal anomaly and partial segmental IH has not been described. Herein, we present 2 patients with talipes equinovarus and an ipsilateral IH.

## Case presentations

### Case 1

**Fig 1:**
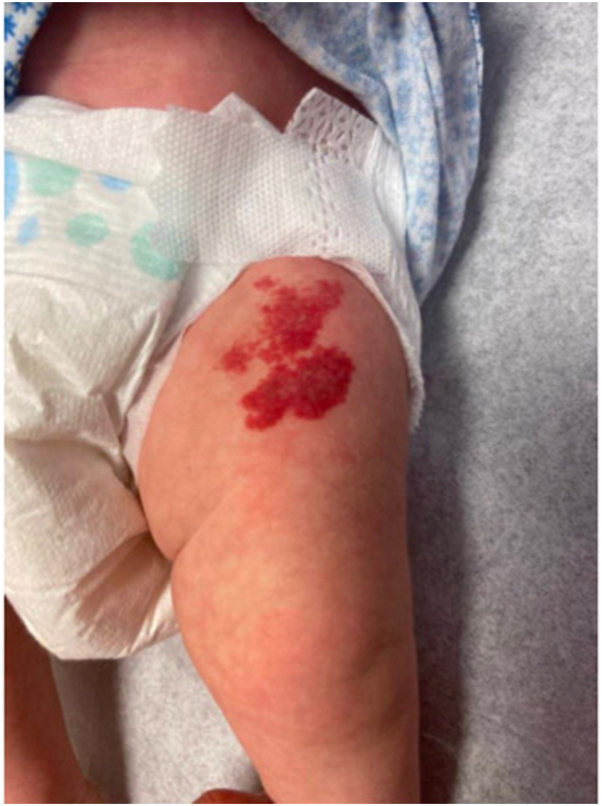
36-day-old female with a partially segmental infantile hemangioma involving the left proximal thigh.

A 36-day-old female presented for evaluation of 2 superficial IHs. The patient was born at term with delivery complicated by an emergency cesarean section due to decreasing fetal heart tones. A left club foot of unclear etiology was noted at birth. On examination, she had one localized bright red thin papule on her mid-back and a larger, partially segmental red lobulated thin plaque (less than 5 cm) involving the left proximal thigh (Fig 1). There was a low concern for LUMBAR syndrome due to the size being less than 5 cm and the nonsegmental nature of the lesion. However, further work-up was obtained due to the lesion being ipsilateral to her club foot. An ultrasound with Doppler of the entire left lower extremity was completed to evaluate for underlying arterial anomalies. Her workup was unremarkable, and the patient has since had her club foot corrected along with natural involution of her IHs.

### Case 2

**Fig 2:**
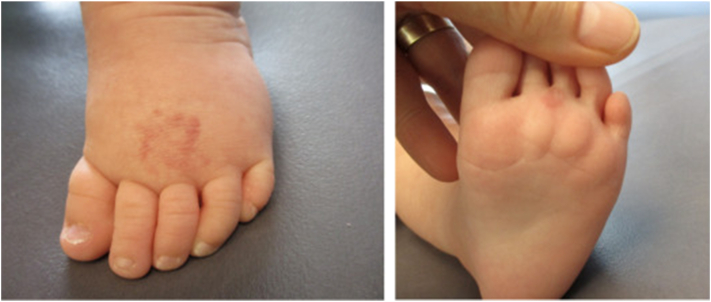
2-month-old male with a multifocal, partial segmental infantile hemangioma of his left distal foot.

A 2-month-old male born at term via uncomplicated vaginal delivery presented for evaluation of an IH on his left foot. He was born with a left club foot of unclear etiology, which had undergone repair, casting, and bracing before evaluation. The examination was notable for a partial segmental light pink thin plaque of his left distal foot, ipsilateral to the limb deformity (Fig 2). There was a similar low concern for LUMBAR syndrome given its focal, partial segmental nature and due to its location on the limb. Given the unknown association between the club foot and hemangioma, however, a left lower extremity ultrasound with Doppler was obtained, which was unremarkable. Additionally, the patient was referred to Neurology for evaluation of developmental anomalies, and further workup with a magnetic resonance imaging brain and entire spine without contrast was negative for central nervous system structural defects or other anomalies.

## Discussion

Per recently published guidelines, the diagnosis of LUMBAR syndrome requires a segmental IH of the lumbosacral, sacrococcygeal, and/or pelvic cutaneous regions plus one additional criterion.^1^ A segmental hemangioma is defined as a large plaque that involves a specific territory, and these lesions are generally known to be at a much higher risk for complications, including underlying congenital anomalies regional to the IH.^2^ These segmental hemangiomas seen in LUMBAR syndrome are also often of “minimal or arrested growth” morphology. IH minimal or arrested growth appears at birth as a telangiectatic patch that develops increased erythema, small papules, or a thin plaque with minimal proliferation; however, they are associated with a high risk of LUMBAR syndrome and the development of ulceration.^1^ The location of an IH may also suggest the risk of underlying anomalies.^2^ For example, a lesion located in the lumbar region is strongly correlated with underlying spinal cord malformations, while those over the sacral region may be associated with urogenital, anorectal, or renal anomalies, although prospective studies are needed to understand this association.^3^ Given the importance of location in potentially guiding further workup, the lower body anatomy has been divided into 4 regions: lumbar T12-L5 intervertebral spaces, sacral area between the anterior iliac crests and perineum/genitals, perineum and genitals, and the lower extremity.^3^ Interestingly, hemangiomas, deemed indeterminate or partial segmental, likely represent a later hit in the embryologic development and have a lower, yet largely unknown, risk of associated anomalies.^1^ In a multicentered study analyzing anogenital IHs, most of which were focal or partial segmental, one in every 16 infants had a concurrent genital anomaly such as a urogenital malformation or myelopathy.^4^ This may support screening for underlying structural anomalies in patients with partial segmental IHs, although all published hemangiomas associated with LUMBAR syndrome are segmental.

Club foot, or talipes equinovarus, is a musculoskeletal birth defect in the United States which occurs in one in every 1000 live births.^5^ While it most commonly is idiopathic, various concomitant abnormalities, such as in extremity vasculature, have been identified.^5^ In a small cohort study, 40% of patients with a unilateral clubfoot had arterial anomalies in the affected limb.^5^ Although it has previously been suggested that nonsegmental IHs on an extremity do not affect limb development, in another study, there were at least 2 cases (8.3%, 2/24) with bony deformities of the lower extremities or pelvis, suggesting that possible underlying arterial anomalies may affect limb development in these cases.^3^ Additionally, IHs extending over the leg have also been associated with limb atrophy, deformity, or leg length discrepancy, and monitoring for this with serial limb measurements and gait is recommended as a child develops.^1^ Although talipes equinovarus is a common musculoskeletal defect, and therefore may be unrelated to the development of an ipsilateral hemangioma, these findings may suggest an association and consideration of further workup.

Finally, it is important to note that LUMBAR syndrome has recently been demonstrated to have significant overlapping findings with additional early embryonic malformation syndromes, such as omphalocele-exstrophy-imperforate anus-spinal defects complex. The omphalocele-exstrophy-imperforate anus-spinal defects complex is included under recurrent constellations of embryonic malformations, which represent an overlapping spectrum of rare disorders of caudal dysgenesis.^6^ Children with this condition also have orthopedic complications, with an incidence of talipes equinovarus being 9% to 27% in one cohort studied.^7^ Therefore, this may suggest that all of these conditions may exist on a spectrum with shared underlying pathogenesis rather than separate disorders, which may further support underlying screening investigation in these patients.^1^

## Conclusion

While it has previously been suggested that nonsegmental IHs involving the lower extremities do not affect limb development, the ipsilateral nature of the hemangioma in these cases to their talipes equinovarus raises the possibility of a connection. Although segmental or partial segmental IHs isolated to the limb do not need to undergo evaluation for LUMBAR syndrome (unlike those found in the lumbosacral, sacrococcygeal, and/or pelvic cutaneous regions), it may be prudent to consider further workup using LUMBAR syndrome guidance to rule out additional underlying anomalies in the setting of an ipsilateral musculoskeletal anomaly, such as talipes equinovarus. Additional prospective studies are needed to further investigate this possible connection and determine an appropriate workup.

## Conflicts of interest

Dr Lalor is a speaker for Krystal Biotech. Dr Foschi has no conflicts of interest to declare.

## References

[1] Metry D., Copp H.L., Rialon K.L. (2024). Delphi consensus on diagnostic criteria for LUMBAR syndrome. J Pediatr.

[2] Yu X., Zhang J., Wu Z. (2017). LUMBAR syndrome: a case manifesting as cutaneous infantile hemangiomas of the lower extremity, perineum and gluteal region, and a review of published work. J Dermatol.

[3] Iacobas I., Burrows P.E., Frieden I.J. (2010). LUMBAR: association between cutaneous infantile hemangiomas of the lower body and regional congenital anomalies. J Pediatr.

[4] Arnold J.D., Yoon S., Shah N. (2023). Characteristics and complications of anogenital infantile hemangiomas: a multicenter retrospective analysis. JAAD.

[5] Merrill L., Gurnett C.A., Siegel M. (2011). Vascular abnormalities correlate with decreased soft tissue volumes in idiopathic clubfoot. Clin Orthop Relat Res.

[6] Barrios L., Chamlin S., Keppler-Noreuil K.M. (2024). LUMBAR syndrome-OEIS complex overlap: a case series and review. Am J Med Genet A.

[7] Keppler-Noreuil K.M. (2001). OEIS complex (omphalocele-exstrophy-imperforate anus-spinal defects): a review of 14 cases. Am J Med Genet.

